# Development of a New Hybrid Biodegradable Drug-Eluting Stent for the Treatment of Peripheral Artery Disease

**DOI:** 10.1155/2016/6915789

**Published:** 2016-11-24

**Authors:** Jung-Hee Lee, Soon-Joong Kim, Se-Il Park, Young-Guk Ko, Donghoon Choi, Myeong-Ki Hong, Yangsoo Jang

**Affiliations:** ^1^Division of Cardiology, Yeungnam University Medical Center, Yeungnam University College of Medicine, Daegu, Republic of Korea; ^2^Division of Cardiology, Department of Internal Medicine, Yonsei University College of Medicine, Seoul, Republic of Korea; ^3^Cardiovascular Research Institute, Yonsei University College of Medicine, Seoul, Republic of Korea; ^4^Cardiovascular Product Evaluation Center, Yonsei University College of Medicine, Seoul, Republic of Korea; ^5^Severance Biomedical Science Institute, Yonsei University College of Medicine, Seoul, Republic of Korea

## Abstract

This study aimed to develop a new biodegradable stent for peripheral artery disease (PAD) that could provide sufficient radial force to maintain long-term patency and flexibility. All self-expandable hybrid biodegradable stents were designed by using a knitting structure composed of poly-L-lactic acid (PLLA) and nitinol. Four different types of stents were implanted in 20 iliac arteries in 10 mini pigs as follows: a bare-metal stent (BMS) (group 1, *n* = 5), a drug-free hybrid stent (group 2, *n* = 5), a 50% (50 : 100,* w*/*w*) paclitaxel (PTX)/poly-lactide-*co*-glycolic acid (PLGA; fast PTX-releasing form) hybrid stent (group 3, *n* = 5), and a 30% (30 : 100,* w*/*w*) PTX/PLGA (slow PTX-releasing form) hybrid stent (group 4, *n* = 5). We performed follow-up angiography and intravascular ultrasonography (IVUS) at 4 and 8 weeks. In a comparison of groups 1, 2, 3, and 4, less diameter stenosis was observed in the angiographic analysis for group 4 at the 4-week follow-up (19.0%  ±  12.7% versus 39.3%  ±  18.1% versus 46.8%  ±  38.0% versus 4.8%  ±  4.2%, resp.; *p* = 0.032). IVUS findings further suggested that the neointima of the patients in group 4 tended to be lesser than those of the others. Our new biodegradable 30% PTX/PLGA (slow-releasing form) stent showed more favorable results for patency than the other stent types.

## 1. Introduction

Biodegradable stents appear to be one of the most promising tools in the field of endovascular intervention, offering numerous potential benefits over permanent implants for the treatment of cardiovascular disease. Percutaneous transluminal angioplasty with primary stenting for peripheral artery disease (PAD) can result in technical success and clinical benefits [1]. Aside from improving flexibility, endovascular stenting using nitinol metal stents may avoid problems such as early elastic recoil, residual stenosis, and flow-limiting dissection after balloon angioplasty [2–4].

Despite the superiority of nitinol metal stents, several major concerns emerged regarding their metallic components, including stent fracture, late stent thrombosis, and late restenosis. The superficial femoral artery is a harsh environment for a metallic stent, as mechanical forces such as bending, torsion, compression, and elongation occur during everyday activities [5]. Hence, the use of biodegradable stents in PAD has previously been investigated [6, 7]. These biodegradable stents have several limitations, including vascular inflammation, lack of radial force for long-term patency, and acute recoiling after stent implantation. However, only few clinical data are available regarding the use of biodegradable stents for peripheral disease or experimental trials aimed at developing new models of biodegradable stents. Thus, the development of a new biodegradable stent for use in PAD is necessitated. The primary aim of this study was to develop a new biodegradable stent for PAD that could provide sufficient radial force for the maintenance of patency, flexibility to vessel geometry, and long-term patency by inhibiting intimal hyperplasia.

## 2. Materials and Methods

### 2.1. Stent Design

**Figure 1:**
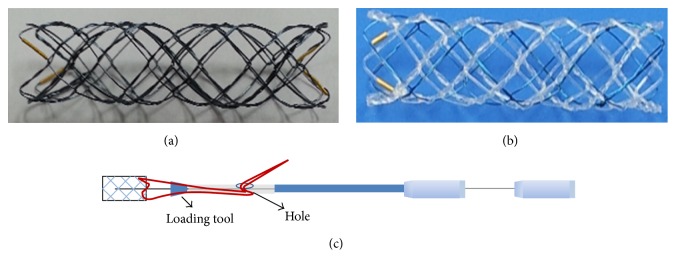
Stent design. (a) Conventional bare-metal stent. (b) Poly-L-lactic acid (PLLA) and nitinol knitting structure of the hybrid biodegradable stent. (c) Self-expandable delivery system of the hybrid biodegradable stent.

The hybrid biodegradable stents were designed to be flexible and self-expanding by using a knitting structure that comprised poly-L-lactic acid (PLLA) with a strut thickness of 225 *μ*m and nitinol (Figure 1). By using a dipping method, we loaded paclitaxel (PTX) to the PLLA/nitinol composite stent. Dip coating was performed by immersing the PLLA/nitinol composite stent in a coating solution (12% poly-lactide-*co*-glycolic acid [PLGA] + 2 : 3* v*/*v* ethanol : dimethyl sulfoxide [DMSO]). For the purpose of achieving optimal PTX levels, we used multiple dipping methods. By controlling the dipping time or contents of the solvent, we regulated the PTX level loaded on a PLLA/nitinol composite stent. The amount of PTX released by each sample was measured by using high-performance liquid chromatography (1% SDS in PBS, 37°C). After analyzing variable PTX loading methods, we chose to use two types of drug-eluting PLLA/nitinol composite stents, a 50% (50 : 100,* w*/*w*) PTX/PLGA (fast PTX-releasing form) hybrid stent, and a 30% (30 : 100,* w*/*w*) PTX/PLGA (slow PTX-releasing form) hybrid stent.

### 2.2. Stent Implantation Procedure

This study was approved by the Yonsei University Institutional Animal Care and Use Committee. Experiments were planned by using 20 common iliac arteries in 10 mini pigs. All the animals received 100 mg of aspirin and 300 mg of clopidogrel at least 12 hours before the procedure. All the animals received humane care in compliance with the Animal Welfare Act and “The Guide for the Care and Use of Laboratory Animals” formulated by the Institute of Laboratory Animal Research [8]. Anesthesia was induced by using intramuscular injections of ketamine (20 mg/kg) and xylazine (2 mg/kg). After adequate systemic anesthesia was attained, the animals were placed in the supine position under mechanical ventilation and isoflurane (1-2%) was delivered by using a precision vaporizer and circle absorption breathing system, with periodic arterial blood gas monitoring. After surgical exposure of the carotid artery, an arteriotomy of the carotid artery was performed under sterile conditions and a 6-Fr vascular access sheath was inserted. During the procedure, vital signs were consistently monitored by using surface electrocardiography. Prior to the procedure, heparin (150 units/kg) was injected to maintain an activated clotting time of ≥250 seconds. Based on quantitative imaging analyses, oversized balloon inflation with a 1.3 : 1.0 balloon artery ratio was applied twice for a period of 30 seconds at a time in each iliac artery. After balloon injury, four different types of stents were randomly implanted in 20 common iliac arteries under fluoroscopic guidance as follows: a bare-metal stent (BMS; group 1, *n* = 5), a drug-free hybrid stent (group 2, *n* = 5), a 50% PTX/PLGA (fast PTX-releasing form) hybrid stent (group 3, *n* = 5), and a 30% PTX/PLGA (slow PTX-releasing form) hybrid stent (group 4, *n* = 5). Operators were blinded to the types of stent used for each procedure. After implantation, conventional angiography was performed, and the carotid arteries were repaired by using suture material suited until subsequent use of the carotid arteries. All the animals received 100 mg of aspirin and 75 mg of clopidogrel daily after stent implantation. The animals were fed a regular diet throughout the duration of the study. Follow-up angiography was performed, and gray-scale intravascular ultrasonographic (IVUS) examinations were conducted in the region of the inserted stent at 4 and 8 weeks after stent implantation. A 2.9-Fr IVUS imaging catheter (Eagle Eye, Volcano Corp, Rancho Cordova, CA) with a 20-MHz phased-array transducer was used. Follow-up IVUS imaging was not performed where angiography had shown total in-stent occlusion. After 8 weeks, the animals were euthanized and the iliac arteries were harvested.

### 2.3. Quantitative Coronary Angiography and Intravascular Ultrasonographic Analysis

Quantitative angiography analyses were performed by using an offline computerized quantitative coronary angiographic system (CASS System, Pie Medical Imaging, Maastricht, The Netherlands) in an independent core laboratory (Cardiovascular Research Center, Seoul, Republic of Korea). The minimal lumen diameter (MLD) with diameter stenosis and the reference diameter (RD) of the treated iliac vessels were measured. The percentage of diameter stenosis was calculated by using the following formula: percent diameter = [(mean RD − MLD)/mean RD] × 100, mean RD = (proximal reference vessel diameter + distal vessel diameter)/2.

Conventional gray-scale quantitative IVUS analyses were performed according to the criteria of the clinical expert consensus document on IVUS and included the external elastic membrane (EEM), lumen, plaque, and media (P&M; P&M = EEM minus lumen) volumes [9]. Cross-sectional IVUS images were analyzed at 1 mm intervals. All IVUS images were analyzed at the core laboratory (Cardiovascular Research Center, Seoul, Republic of Korea) by analysts who were blinded to the treatment and procedures performed in each animal.

### 2.4. Histological Analysis

All the animals were euthanized under anesthesia after the 8-week follow-up images had been acquired. Immediately after the iliac arteries were harvested, the stented vascular segments were fixed for 24 hours by using 4% formaldehyde. After dehydration, the samples were embedded in a glycol methacrylate (GMA) polymerization solution (Technovit 7200VLC, Heraeus Kulzer Gmbh, Germany). Each stented segment was cut proximally, medially, and distally by using an EXAKT saw (EXAKT Apparatebau, Germany); stained; dried; and glued onto EXAKT slides; and polished down by using an EXAKT-polish machine (EXAKT 400 CS, EXAKT Apparatebau, Germany). Data analysis was performed by using a microscope (GE-OEC Series 9800, USA) and its corresponding imaging software (Sigmascan Pro, Systat Software Inc., USA). All embedded sections were stained with hematoxylin-eosin.

### 2.5. Evaluation of Inflammatory and Vessel Injury Scores

Inflammatory scores were defined as follows: 0, no inflammatory cells surrounding the strut; 1, light, noncircumferential lymphohistiocytic infiltrate surrounding the strut; 2, localized, noncircumferential, moderate-to-dense cellular aggregate surrounding the strut; and 3, circumferential dense lymphohistiocytic cell infiltration of the strut [10, 11]. The vessel injury score was graded as follows: 0, internal elastic lamina intact; 1, internal elastic lamina lacerated; 2, internal elastic lacerated; and 3, external elastic lamina lacerated [10].

### 2.6. Statistical Analysis

Statistical analysis was performed by using SPSS (Version 20.0.0, IBM, Armonk, NY, USA). Data were expressed as number (%) or mean ± standard deviation or median (interquartile range). Continuous variables were compared by using one-way analysis of variance or the Kruskal-Wallis test. Abnormally distributed continuous variables were compared by using the Mann–Whitney *U* test. Comparisons of categorical data were performed by using chi-square or Fisher exact test. A Pearson correlation analysis was performed to evaluate the correlation between changes in MLD. A *p* value of <0.05 was considered statistically significant.

## 3. Results and Discussion

### 3.1. Results

**Figure 2:**
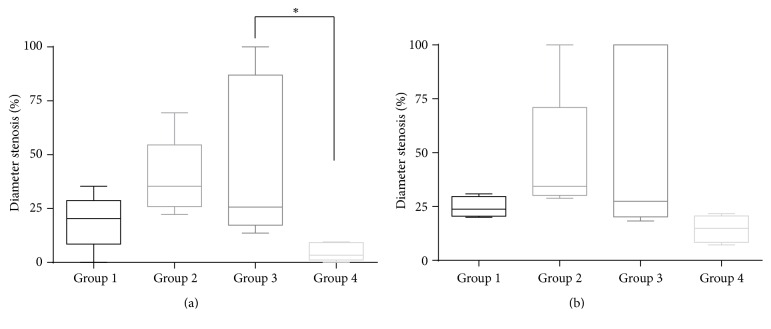
Comparison of diameter stenosis among the groups (^*∗*^ *p* < 0.05). (a) At 4-week follow-up. (b) At 8-week follow-up. G1 (group 1): bare-metal stent (BMS), G2 (group 2): drug-free hybrid stent, G3 (group 3): 50% PTX/PLGA (fast PTX-releasing form) hybrid stent, and G4 (group 4): 30% PTX/PLGA (slow PTX-releasing form) hybrid stent.

**Figure 3:**
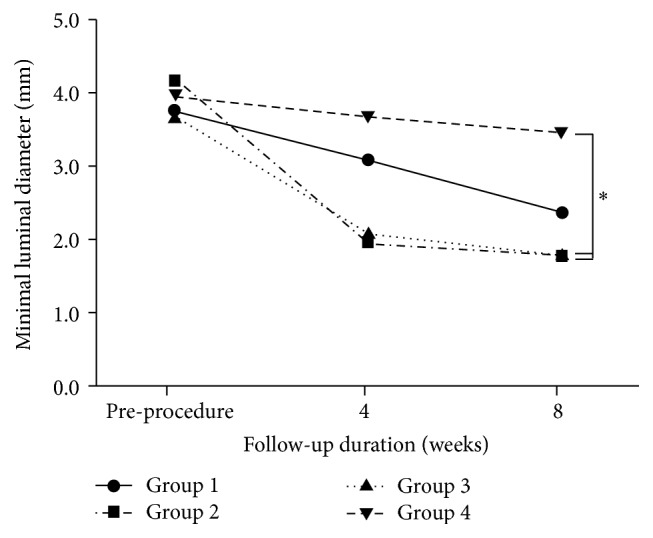
Serial changes in minimal luminal diameter at 4- and 8-week follow-ups (^*∗*^ *p* < 0.05). G1 (group 1): bare-metal stent (BMS), G2 (group 2): drug-free hybrid stent, G3 (group 3): 50% PTX/PLGA (fast PTX-releasing form) hybrid stent, and G4 (group 4): 30% PTX/PLGA (slow PTX-releasing form) hybrid stent.

All the stents were successfully implanted to both common iliac arteries in all the 10 animals. The findings from the quantitative imaging analyses are presented in Table 1. The RDs of the iliac arteries before the procedure, at the 4-week follow-up, or at the 8-week follow-up did not show significant differences. When groups 1, 2, 3, and 4 were compared, group 4 showed less diameter stenosis in the 30% PTX/PLGA (slow PTX-releasing form) hybrid stent at the 4-week follow-up (19.0%  ±  12.7% versus 39.3%  ±  18.1% versus 46.8%  ±  38.0% versus 4.8%  ±  4.2%, resp.; *p* = 0.032, Figure 2). The MLD in group 4 (3.39 ± 0.45 mm) also tended to be greater than that in the other groups at the 8-week follow-up, although the difference was not statistically significant (*p* = 0.108). However, the MLD in group 4 showed more favorable results when compared with those in groups 2 (drug-free hybrid stent) and 3 (50% PTX/PLGA, fast PTX-releasing hybrid stent; Figure 3).

The findings from the IVUS imaging analyses are summarized in Table 2. IVUS imaging was not performed if angiography showed total in-stent occlusion. One lesion in group 3 showed total occlusion at the 4-week follow-up, while one lesion in group 2 and two lesions in group 3 showed total occlusion at the 8-week follow-up. IVUS images obtained at the 4-week follow-up showed that the lumen area in group 4 was significantly larger than that in the other groups. The neointimal area of the group 4 stent also tended to be less than that of the other stents used, although the difference was not statistically significant. IVUS images obtained at the 8-week follow-up showed similar results, although no statistically significant differences were observed in the lumen and neointimal area. The stent area as measured by using IVUS did not show statistically significant changes during the study periods across the groups.

**Figure 4:**
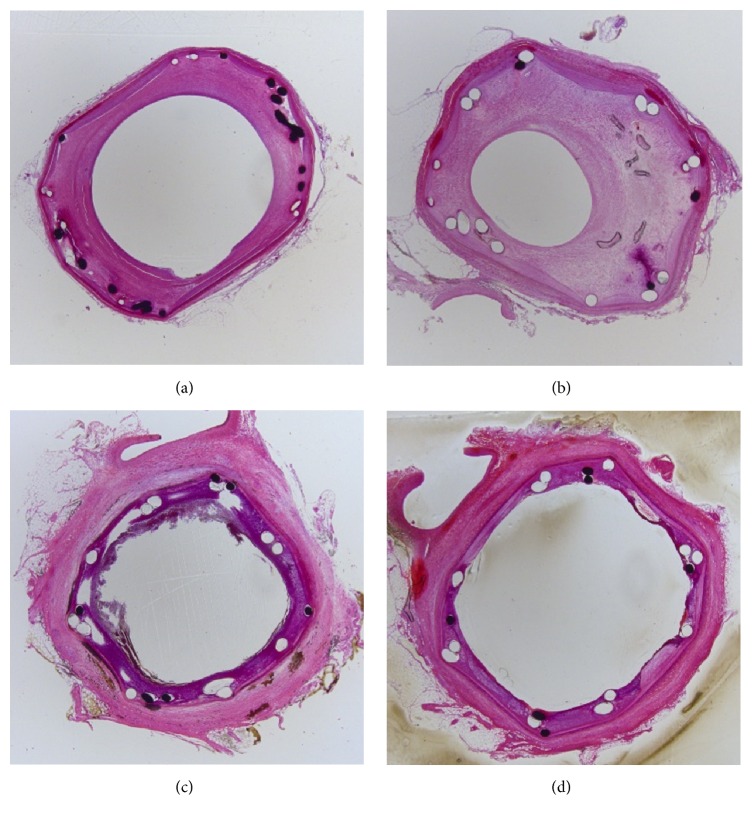
Representative histological images of each stent. (a) A bare-metal stent (group 1). (b) A drug-free hybrid stent (group 2). (c) A 50% PTX/PLGA (fast PTX-releasing form) hybrid stent (group 3). (d) A 30% PTX/PLGA (slow PTX-releasing form) hybrid stent (group 4).

The findings from the histopathological assessment are presented in Table 3. All group 1 (*n* = 5) and group 4 (*n* = 5) stents showed a low-grade inflammatory score (0-1) 8 weeks after stent implantation. Furthermore, all the group 4 stents showed a low-grade vessel injury score (0-1) at 8 weeks after the procedure.  Figure 4 shows representative histological images of each stent type.

### 3.2. Discussion

The major findings of this study were as follows: (1) our new hybrid biodegradable stent designed to be self-expanding with a knitting structure composed of PLLA and nitinol was easy to deploy without complication and (2) the 30% PTX/PLGA (slow PTX-releasing form) hybrid stent more effectively inhibited neointimal hyperplasia without inflammation and vascular injury compared to the other stent types.

Self-expandable nitinol stents have been developed for the treatment of femoropopliteal disease, and primary nitinol stenting is recommended as a first-line treatment for superficial femoral artery lesions [3, 4, 12]. However, the high rate of in-stent restenosis is a major problem of endovascular treatment with metallic stents [13]. Furthermore, previous case reports have shown that nitinol stents have major limitations attributable to their metallic components, such as stent fracture or crushed stents [14, 15]. Biodegradable scaffolds have clear advantages over metallic stents, not only for coronary intervention but also for peripheral artery revascularization, including lower incidence of adverse events such as thrombotic stent reocclusion and stent fracture [16, 17]. PLLA consists of a crystalline component of semicrystalline polymer and is widely used in biodegradable scaffolds [16, 18]. In general, in vivo studies that investigated bioresorbable scaffolds have shown an initial reduction in molecular weight, a decrease in radial support at about 6 months, loss in mass starting at 12 months, and subsequent completion at 24 months [16, 18, 19]. However, radial support for just 6 months may not be sufficient for peripheral artery intervention, considering that these arteries encounter a significant amount of compression, torsion, extension, and bending. In the present study, IVUS findings demonstrated that our new biodegradable stent did not show significant recoil during the 8-week follow-up period. This new biodegradable stent for the treatment of PAD is expected to provide superior radial support when compared with conventional bioresorbable scaffolds consisting only of PLLA.

Several recent studies have suggested that clinical outcomes in patients treated with everolimus-eluting bioresorbable scaffolds for coronary artery revascularization are within the range for noninferiority when compared with drug-eluting metallic stents [20–23]. Werner et al. previously reported their experience with the use of a biodegradable balloon expandable stent composed of PLLA, the Igaki-Tamai biodegradable stent, for the treatment of de novo lesions in the femoral artery [6]. The authors demonstrated excellent short-term results; however, sustainable luminal patency over time and inflammatory reaction continue to be concerns. That bioabsorbable polymer is more likely to be associated with an inflammatory reaction compared to a nitinol-based BMS. The results of the present study found that the 30% PTX/PLGA (slow PTX-releasing form) hybrid stent showed a low level of inflammation comparable with that with BMS. Therefore, our new hybrid biodegradable drug-eluting stent for the treatment of PAD has potential clinical benefits by reducing the inflammatory reaction after stent implantation. Furthermore, we expect that our new hybrid biodegradable stent will enable vascular surgeons to easily perform surgical procedures for stenting lesions after stent restenosis.

The present study demonstrated that the 30% PTX/PLGA (slow PTX-releasing form) hybrid stent in group 4 was more strongly associated with inhibition of in-stent neointimal hyperplasia than the drug-free hybrid stent (group 2) and the 50% PTX/PLGA (fast PTX-releasing form) hybrid stent (group 3). A study that evaluated the kinetics of paclitaxel release on the neointima showed that the longer-releasing paclitaxel-eluting stent had the best results in terms of inhibition of in-stent neointimal hyperplasia [24]. Our findings also suggest that the duration of paclitaxel release had a significant impact on the suppression of in-stent neointimal hyperplasia. While clear reasons for these results are uncertain, we hypothesized that the inhibition of the proliferative reaction requires a minimum period.

This study had several limitations. First, this study was not based on the atherosclerotic porcine peripheral artery model. Therefore, its results require caution when applied in a clinical setting for the treatment of atherosclerotic PAD. Second, the statistical power of our findings was not sufficient because of the relatively small sample size and short study period. However, angiography results were statistically significant at the 4-week follow-up and IVUS findings were similar to the quantitative imaging analysis results. Third, we did not fully evaluate the total occluded stent segments by using IVUS. However, we analyzed all histopathological assessment results regardless of total occlusion.

## 4. Conclusion

Our new self-expandable, biodegradable 30% PTX/PLGA (slow-releasing form) stent was successfully implanted to all iliac arteries and showed the most favorable results for patency and safety when compared with the other stent types. These findings strongly suggest the need for further, large-scale, and long-term experimental study aimed at clinical application. However, our study provides new concepts for developing a new biodegradable stent for the treatment of PAD.

## Figures and Tables

**Table 1:** Quantitative imaging analyses.

	Group 1 (*n* = 5)	Group 2 (*n* = 5)	Group 3 (*n* = 5)	Group 4 (*n* = 5)	*p*
Preprocedure RD (mm)	3.79 ± 0.50	4.20 ± 0.38	3.71 ± 0.52	4.00 ± 0.60	0.453
*4-week follow-up*					
RD (mm)	3.72 ± 0.63	3.30 ± 1.09	3.63 ± 0.67	3.85 ± 0.82	0.778
MLD (mm)	3.01 ± 0.52	1.97 ± 0.81	2.11 ± 1.66	4.06 ± 0.39	0.013
DS (%)	19.0 ± 12.7	39.3 ± 18.1	46.8 ± 38.0	4.8 ± 4.2	0.032
*8-week follow-up*					
RD (mm)	3.90 ± 0.39	3.39 ± 0.81	3.51 ± 0.54	4.02 ± 0.29	0.242
MLD (mm)	3.03 ± 0.46	1.80 ± 1.08	1.81 ± 1.67	3.39 ± 0.45	0.108
DS (%)	24.6 ± 4.8	47.3 ± 29.8	53.6 ± 42.5	14.6 ± 6.3	0.172

Group 1: bare-metal stent (BMS), group 2: drug-free hybrid stent, group 3: 50% PTX/PLGA (fast PTX-releasing form) hybrid stent, and group 4: 30% PTX/PLGA (slow PTX-releasing form) hybrid stent.

RD: reference diameter, MLD: minimal luminal diameter, and DS: diameter stenosis.

**Table 2:** Intravascular ultrasonographic findings from the maximum neointimal site at 4- and 8-week follow-ups.

	Group 1 (*n* = 5)	Group 2 (*n* = 5)	Group 3 (*n* = 4)	Group 4 (*n* = 5)	*p*
*4-week follow-up*					
Stent area, mm^2^	23.0 ± 1.1	20.8 ± 2.7	22.6 ± 5.0	24.9 ± 2.5	0.218
Lumen area, mm^2^	12.9 ± 6.4	8.4 ± 3.3	10.2 ± 5.2	18.2 ± 4.3	0.037
Neointimal area, mm^2^	10.0 ± 5.8	12.4 ± 3.3	12.6 ± 4.7	6.8 ± 3.7	0.196
Percentage of NIH (%)	44.3 ± 25.9	59.8 ± 14.7	56.1 ± 17.4	27.4 ± 15.3	0.072
*8-week follow-up*	(*n* = 5)	(*n* = 4)	(*n* = 3)	(*n* = 5)	
Stent area, mm^2^	23.1 ± 1.3	19.2 ± 3.4	23.6 ± 6.1	23.6 ± 3.1	0.248
Lumen area, mm^2^	9.5 ± 2.7	7.5 ± 3.7	12.5 ± 3.9	12.9 ± 2.2	0.072
Neointimal area, mm^2^	13.6 ± 1.9	11.7 ± 1.6	11.2 ± 2.6	10.7 ± 3.8	0.379
Percentage of NIH (%)	59.1 ± 10.4	62.6 ± 14.8	47.5 ± 4.9	44.5 ± 12.0	0.104

Group 1: bare-metal stent (BMS), group 2: drug-free hybrid stent, group 3: 50% PTX/PLGA (fast PTX-releasing form) hybrid stent, and group 4: 30% PTX/PLGA (slow PTX-releasing form) hybrid stent.

NIH: neointimal hyperplasia.

**Table 3:** Histopathological assessment of porcine iliac arteries 8 weeks after stenting.

	Group 1 (*n* = 5)	Group 2 (*n* = 5)	Group 3 (*n* = 5)	Group 4 (*n* = 5)	*p*
*Inflammatory score, n (%)*					0.172
0-1	5 (100)	3 (60)	3 (60)	5 (100)	
2-3	0 (0)	2 (40)	2 (40)	0 (0)	
*Vessel injury score, n (%)*					0.414
0-1	3 (60)	3 (60)	3 (60)	5 (100)	
2-3	2 (40)	2 (40)	2 (40)	0 (0)	

Group 1: bare-metal stent (BMS), group 2: drug-free hybrid stent, group 3: 50% PTX/PLGA (fast PTX-releasing form) hybrid stent, and group 4: 30% PTX/PLGA (slow PTX-releasing form) hybrid stent.
